# Establishment and application of a new diagnostic definition of metabolic syndrome in the Shantou region of southern China

**DOI:** 10.1038/srep22210

**Published:** 2016-02-23

**Authors:** Zan Ding, Fuhua Pi, Shengchao Zhang, Wenya Dong, Ye Wen, Jiang Wu, Qingying Zhang

**Affiliations:** 1 Department of Preventive Medicine, Shantou University Medical College, Shantou, Guangdong 515041, China; 2 Department of Sports, Shantou University Medical College, Shantou, Guangdong 515041, China; 3 Shenzhen Baoan District Central Hospital, Shenzhen, Guangdong 518102, China

## Abstract

The existing definitions of metabolic syndrome (MetS) may not be fully appropriate for the Shantou population because of ethnic and regional differences. We sought to establish a 95% multivariate medical reference range (MMRR) model for diagnosing MetS in Shantou adults and to evaluate the prevalence of MetS by the MMRR, JCDCG (the Chinese Guidelines), and International Diabetes Federation (IDF) criteria. A total of 4,580 participants were recruited in Shantou, southern China. We developed a MMRR model based on the combinatorial indicatrixes method for three categorized indicatrixes. According to the developed MMRR criteria, men (women) in Shantou have MetS by meeting 3 or more of the following: waist circumference ≥89 (81) cm; triglycerides level ≥1.73 (1.64) mmol/L; high-density lipoprotein cholesterol level ≤1.07 (1.05) mmol/L; blood pressure ≥138/89 (136/85) mmHg; and fasting plasma glucose ≥5.8 (5.7) mmol/L. The agreement of the MMRR with JCDCG and IDF criteria was “substantial” (both *κ* > 0.68), but the recommended reference values and proportion of individual components of MetS defined by the 3 criteria differed. The population-based MMRR criteria may be appropriate for diagnosing MetS in Shantou population and the model might be useful for generalization to other geographic regions.

Metabolic syndrome (MetS) is a constellation of metabolic disorder components, including obesity (particularly central obesity), dyslipidemia (elevated level of triglycerides [TG] and/or lowered level of high-density lipoprotein cholesterol [HDL-C]), hypertension or hyperglycaemia^1^,^2^; it is associated with the development of type 2 diabetes mellitus^3^,^4^,^5^, cardiovascular events^6^,^7^,^8^,^9^, and death^10^,^11^.

Several definitions of MetS have been proposed by different organizations for clinical practice or research. In 1999, the World Health Organization proposed the first definition^12^, and in 2001, the National Cholesterol Education Program Expert Panel formulated a working definition (NCEP-ATPIII)^13^,^14^. In 2005, the American Heart Association and National Heart, Lung, and Blood Institute updated the NCEP-ATPIII definition for the diagnosis and management of MetS in adults^1^. During the same period, the International Diabetes Federation (IDF) released a clinically accessible worldwide uniform definition of MetS that could be used by any physician in any country, with ethnic-specific central obesity criteria as a prerequisite for diagnosis of MetS^2^,^15^,^16^. In 2007, the Chinese Joint Committee for Developing Chinese Guidelines on Prevention and Treatment of Dyslipidemia in Adults (JCDCG) proposed another definition of MetS, focusing on metabolic disturbance in the Chinese population 17; limited information is available about the prevalence of MetS by the JCDCG definition^18^. Despite differences in cut-off points for metabolic disorder components, the core components (obesity, dyslipidemia, hypertension, and glucose intolerance) among these definitions are similar, but the relative importance of the core components differs.

The population of the Shantou region in southern China was approximately 13.94 million (7.06 million men) in 2010. People who reside in this area still retain their own dialect and have distinct dietary habits, lifestyles, and physical activity^19^. For example, oolong tea is the most widely consumed beverage in the Shantou region, and long-term oolong tea consumption is associated with reduced risk of dyslipidemia (reducted TG level and elevated HDL-C level)^19^. In addition, systolic blood pressure (SBP), diastolic blood pressure (DBP), body weight, body mass index (BMI), waist circumference (WC), and fasting plasma glucose (FPG) are lower in people from southern than northern China^20^.

Ethnic and regional differences likely exist among populations across the world^2^,^21^,^22^, and the various existing definitions of MetS may not be appropriate for the Shantou population. The diagnostic definition of MetS for Shantou adults should be based on the characteristics of metabolic disturbance in the Shantou region. This study aimed to establish and evaluate a 95% multivariate medical reference range (MMRR) model that may improve the sensitivity and specificity of MetS diagnosis and simplify the clinical diagnosis of MetS in the Shantou region. An additional aim was to estimate the prevalence of MetS among Shantou adults by comparing the developed MMRR diagnostic definition and the JCDCG and IDF definitions.

## Methods

### Study subjects

We conducted a population-based study of MetS in the Shantou region of southern China from January 2010 to June 2013 with 4,580 participants (3,062 men) aged 18 to 90 years. All participants were ≥18 years old, of Han nationality, and were Shantou natives who had been living in the area for at least 5 years before the survey. Immigrants, pregnant women, and people with serious heath problems (e.g., cancer, heart disease, and stroke) were excluded. Participants were randomly recruited from the chronic disease-monitoring sites in the health care centres of the First Affiliated Hospital of Shantou University Medical College and the Chaonan Minsheng Hospital of Shantou.

This study complied with the Declaration of Helsinki and was approved by the Ethics Committee of the First Affiliated Hospital of Shantou University Medical College and Chaonan Minsheng Hospital. Written informed consent was obtained from all participants.

### Data collection

WC was measured by a trained physician who used a non-elastic tape at the horizontal plane between the inferior costal margin and the iliac crest on the midaxillary line, with subjects standing relaxed and wearing one layer of clothing. The same physician used a mercury sphygmomanometer to record blood pressure (BP) to the nearest 1 mmHg on the right upper limb with subjects in the sitting position. BP readings were taken at least 30 sec apart after participants had rested for 5 to 10 min. SBP and DBP were averaged from 3 measurements. Fasting blood samples were obtained by venipuncture in the early morning and all assessments were performed in the clinical laboratory at the hospitals. Blood plasma TG, HDL-C, and FPG concentrations were measured with the same batch reagents by an enzymatic method with a Beckman auto-analyzer (Beckman DXC800, Beckman Coulter, Inc.).

### Definitions of MetS by the JCDCG and IDF criteria

According to the JCDCG definition^17^,^18^, MetS is diagnosed with at least 3 of the 5 component risk factors (elevated WC, TG level, BP [SBP and/or DBP], and FPG, or reduced HDL-C level) (Table 1). With IDF diagnostic criteria^2^,^16^, MetS is diagnosed in a Chinese person with central obesity (increased WC) plus at least 2 other risk factors (Table 1). These criteria include 6 individual components of MetS (WC, TG, HDL-C, SBP, DBP, and FPG), which can be divided into 3 categories: WC, TG, and HDL-C for type I; SBP and DBP for type II; and FPG for type III.

### Establishing a new diagnostic definition of MetS

The 95% univariate reference interval is commonly used in clinical studies as the standard tool for interpreting medical data^23^,^24^. However, a well-known statistical dilemma appears with the simultaneous use of >1 univariate reference interval^23^,^25^. The proportion of observations classified as abnormal increases with the 95% multiple univariate reference intervals^25^. For instance, a healthy person has an *a priori* 5% probability of abnormal values; the same person evaluated with 6 independent 95% univariate reference intervals will have an *a priori* probability of 1–0.95^6^, or 26.5% probability abnormal values for all intervals. Thus, the probability of false-positive observations increases with number of univariate intervals, and one way to deal with this dilemma is the use of a multivariate reference model, which decreases the probability of both false-positive and false-negative observations^23^,^25^. The classification of MetS requires meeting several individual components; therefore, the simultaneous interpretation of multiple risk factors for MetS should involvet a multivariate reference model (e.g., the 95% MMRR model) but not multiple univariate reference intervals from a statistical point of view.

To establish a new diagnostic definition of MetS for the Shantou population, we randomly selected 800 “normal” participants (400 men and 400 women) from our sample during January to December in 2010, normal referring to subjects without hypertension, type 2 diabetes, and dyslipidaemia history and not taking medications. The diagnostic definition was established separately for men and women.

In our study, a novel method, the “combinatorial indicatrixes method for three categorized indicatrixes”, was developed by the authors for the 95% MMRR model, a multivariate reference model. The 6 component risk factors, or indicatrixes, of MetS (WC, TG, HDL-C, SBP, DBP, and FPG) could be grouped into 3 categorized indicatrixes: type I with 3 indicatrixes (WC, TG, and HDL-C) (n_1_ = 3), type II with 2 indicatrixes (SBP and DBP) (n_2_ = 2), and type III with 1 indicatrix (FPG) (n_3_ = 1), for n_1_, n_2_, and n_3_ indicatrixes for types I, II, and III, respectively. We assumed that each of the risk factors had the same *a priori* probability of being classified as abnormal, with abnormal rates labeled as *q*. A cut-off percentile (in this study, 95%) was selected to differentiate multivariate “normal” from “abnormal” results. MetS was defined as any of the following 4 situations: (1) type I with *h* abnormal indicatrixes (3 ≤ *h* ≤ n_1_); (2) type I with *h* abnormal indicatrixes (2 ≤ *h* < 3) and type III with *k* abnormal indicatrixes (0 < *k* ≤ n_3_); (3) type I with *h* abnormal indicatrixes (2 ≤ *h* < 3) and type II with *j* abnormal indicatrixes (0 < *j* ≤ n_2_); (4) type I with *h* abnormal indicatrixes (0 < *h* < 2), type II with *j* abnormal indicatrixes (0 < *j* ≤ n_2_), and type III with *k* abnormal indicatrixes (0 < *k* ≤ n_3_). The italics letters *h*, *j*, and *k* are the number of abnormal indicatrixes for types I, II, and III, respectively. Therefore, the 95% MMRR model for diagnosing MetS in the analysis was as follows:


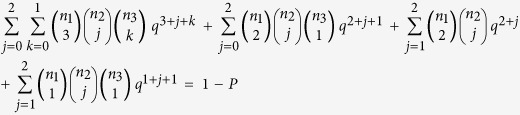


where n_1_ = 3, n_2_ = 2, n_3_ = 1, and *P* = 0.95. Then we obtained a function 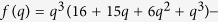

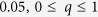
. We used the Newton’s iterative method with the desired accuracy of 10^−10^ to find an approximate real root of the nonlinear equation and then obtained the approximate real root (*q* = 0.140003) after 12 iterations. In other words, each of the 6 risk factors had an *a priori* probability of 14.0% of being classified as abnormal. Using a rank-based method (i.e., the percentile method), we obtained the cut-off points for 6 metabolic disorder components for diagnosing MetS in the Shantou population.

MetS classification by the newly established MMRR criteria required at least 3 of the 5 component risk factors (abnormal WC, TG and HDL-C level, BP, and FPG). In reference to the IDF criteria, the following conditions were also included in the MMRR criteria: treatments for TG or HDL-C abnormality and previously diagnosed hypertension and type 2 diabetes.

### Statistical analysis

The normal distribution of data was determined by the Shapiro–Wilk test. Significance for intergroup differences was assessed by the Mann–Whitney U test for skewed variables and Pearson’s chi-square test with or without Bonferroni correction for categorical variables. A chi-square test for trend was also performed. Categorical variables with percentages and continuous non-normally distributed variables are described with median (interquartile range [IQR]). Study subjects were divided into 6 age groups (18–29, 30–39, 40–49, 50–59, 60–69, and ≥70 years) to standardize the prevalence of MetS and analyze the association of MetS prevalence and age. Age- or sex-standardized prevalence rates were calculated according to the distribution of the Chinese population in the 2010 China population census by the direct standardization method. Cohen’s kappa 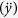 was used to determine agreement between the MMRR and JCDCG, MMRR and IDF criteria; the degree of agreement was interpreted according to Landis and Koch^26^. All statistical analyses involved use of R 2.12.0 (http://www.R-project.org, the R Foundation for Statistical Computing, Vienna, Austria), with the fmsb package used to calculate 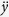 values. Two-tailed tests of significance were reported, with *P* < 0.05 considered statistically significant.

## Results

### Newly established MMRR criteria for MetS

According to the developed MMRR criteria, men (women) in the Shantou region had MetS by meeting ≥3 of the following abnormalities: WC ≥ 89 (81) cm; TG level ≥ 1.73 (1.64) mmol/L or specific treatment for TG abnormality; HDL-C level ≤ 1.07 (1.05) mmol/L or specific treatment for HDL-C abnormality; BP ≥ 138/89 (136/85) mmHg or treatment for previously diagnosed hypertension; and FPG ≥ 5.8 (5.7) mmol/L or previously diagnosed type 2 diabetes (Table 1).

### Sample characteristics

The baseline characteristics of the study population by gender are in Table 2. A total of 4,580 Shantou adults were recruited, with median age 44.0 years (IQR 36.0–57.0). Men were younger than women and had significantly higher WC, TG level, SBP, and DBP but lower HDL-C level (all *P* < 0.001). Men and women did not differ in level of FPG (*P* = 0.277).

### Crude prevalence of MetS

**Figure 1:**
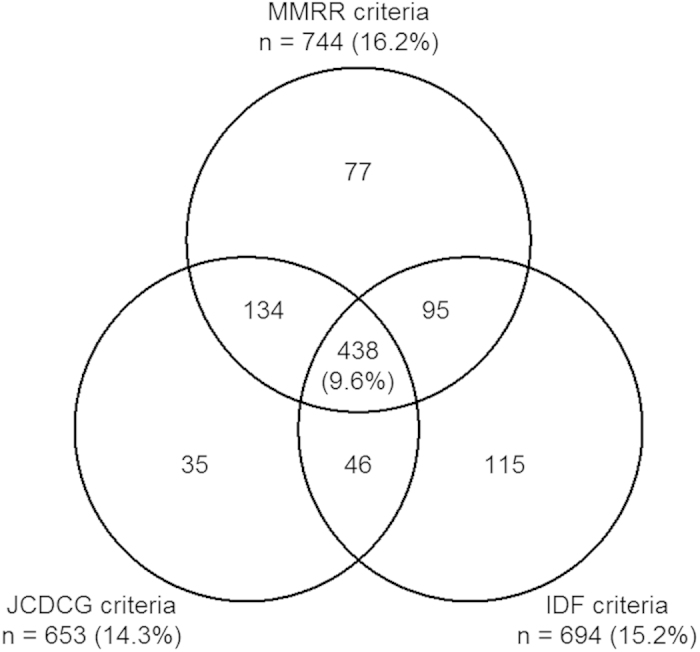
Overall crude prevalence and overlap of metabolic syndrome (MetS) by the JCDCG, MMRR, and IDF criteria among Shantou adults.

Among all subjects, the crude prevalence (and number) with MetS was 16.2% (n = 774), 14.3% (n = 653), and 15.2% (n = 694) by MMRR, JCDCG, and IDF criteria, respectively (Fig. 1). Overall, 438 (9.6%) were identified as having MetS by all 3 definitions, and the number of subjects overlapping between the MMRR and JCDCG and MMRR and IDF criteria was 572 (12.5%) and 533 (11.6%), respectively (Fig. 1). The prevalence of MetS was higher for women than men by the MMRR (17.3% vs 15.7%; *P* = 0.190) and IDF criteria (20.6% vs 12.4%; *P* < 0.001) but higher for men than women by the JCDCG (12.5% vs 15.1%; *P* = 0.018). The proportion of MetS by the 3 criteria differed for men, women (both *P* < 0.001), and total subjects (*P* = 0.030). The prevalence of MetS was higher by the MMRR than JCDCG criteria for women (*P* < 0.001) and total subjects (*P* = 0.008) and by the IDF criteria for men (*P* < 0.001).

### Standardized prevalence of MetS and its individual components

Overall, the age- and sex-standardized prevalence of MetS by the MMRR, JCDCG, and IDF diagnostic criteria was 13.5%, 11.6%, and 13.4%, respectively (*P* = 0.009) (Table 3). The standardized prevalence of MetS by the MMRR (*P* = 0.006) and IDF criteria (*P* = 0.010) was higher than by the JCDCG criteria, with no significant difference between the MMRR and IDF criteria (*P* = 0.903). The age-standardized prevalence of MetS was higher for men than women by the MMRR (14.2% vs 12.9%; *P* = 0.237) and JCDCG criteria (13.8% vs 9.4%; *P* < 0.001) but higher for women than men by the IDF criteria(15.7% vs 11.2%; *P* < 0.001).

Among men, women, and total subjects, the proportions of elevated WC (central obesity), reduced HDL-C level, elevated BP, and elevated FPG by the 3 criteria differed (all *P* < 0.001), with no significant difference in proportion of elevated TG level (all *P* > 0.05) (Table 3). The proportions of central obesity (increased WC), reduced HDL-C level, and elevated FPG was higher by the MMRR than JCDCG criteria but lower for elevated BP. Although the prevalence of MetS was approximately equal by the MMRR and IDF criteria, the proportions of the individual components of MetS by the 2 criteria differed (Table 3). The proportion of men with reduced HDL-C level was greater with the MMRR than the IDF criteria.

According to thresholds for Shantou adults by the new criteria, elevated TG level (30.4%) and WC (28.7%) was the most prevalent component for men and women, respectively; the age-standardized prevalence of elevated TG and reduced HDL-C levels was higher for men than women, but the prevalence of central obesity (WC) was lower (all *P* < 0.001). With MMRR criteria, central obesity, elevated TG level, and elevated BP had a similar proportion among all subjects; 41.6% of all subjects had no individual components of MetS and 0.6% had all 5 metabolic abnormalities (Table 3). Overall, 36.1% of all subjects had elevated BP by both the JCDCG and IDF criteria. The prevalence of reduced HDL-C level by the IDF criteria among men and women was 12.8% and 40.0%, respectively (*P* < 0.001).

**Figure 2:**
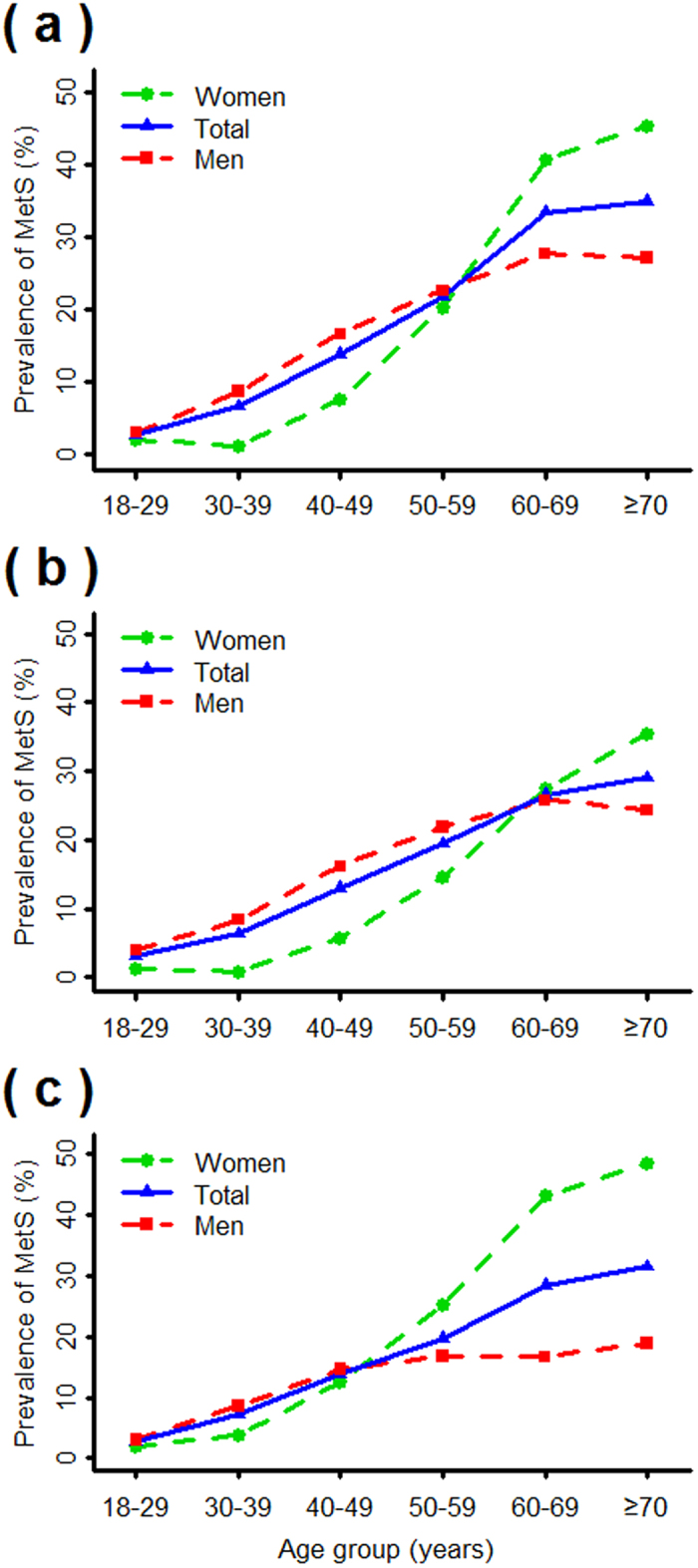
Age-specific percentage of MetS in men, women, and total subjects (sex-standardized) in the Shantou region of southern China by the MMRR (**a**), JCDCG (**b**), and IDF (**c**) criteria.

Regardless of the definition used, the prevalence of MetS greatly increased with increasing age and peaked in the oldest group for men, women, and all subjects (all *P*_trend_ < 0.001); the prevalence was higher for women than men among older adults (age ≥ 60) and lower among younger adults (age ≤ 50) (Fig. 2). By the MMRR criteria, the overall prevalence of MetS increased from 2.7% to 30.2% for subjects 18–29 years old to those > 70 years.

### Agreement between the MMRR and JCDCG and MMRR and IDF criteria

With the JCDCG criteria as a reference, the observed agreement (with or without MetS), sensitivity, specificity, and positive and negative predictive value of the MMRR criteria was 94.48%, 87.60%, 95.62%, 76.88%, and 97.89%, respectively (Table 4). As compared with the IDF criteria, the sensitivity of the new diagnostic criteria was 76.80% and specificity 94.57%. The levels of agreement between the MMRR and JCDCG criteria were “substantial” for women and all subjects (both 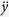 ≥ 0.75, *P* < 0.001) and “almost perfect” for men (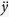 = 0.81, *P* < 0.001). The agreements between the MMRR and IDF criteria were “substantial” for men, women, and all subjects (all 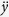 ≥ 0.68, *P* < 0.001) (Table 4).

## Discussion

Considering ethnic and regional differences, the JCDCG or IDF criteria for diagnosing MetS may not be fully appropriate for the Shantou population. To simplify the clinical application of MetS criteria and improve its recognition, we performed a 95% multivariate reference model instead of the 95% multiple univariate reference intervals to establish a new diagnostic definition of MetS for Shantou adults. The 95% MMRR model based on the combinatorial indicatrixes method for three categorized indicatrixes is a valid statistical method for establishing diagnostic criteria for MetS and can be generalized to other geographic regions. We also compared the developed MMRR criteria with the JCDCG and IDF criteria and estimated the prevalence of MetS among adults in the Shantou region of southern China.

The MetS and its components are strongly associated with increased risk of incident type 2 diabetes, with hazard ratios ranging from 4.41 to 7.47^5^. A case–control study in California reported that a strong independent determinant of early-onset coronary artery disease is the presence of MetS without diabetes (adjusted odds ratio [aOR] = 4.9) or with diabetes (aOR = 8.0)^6^. MetS is also a significant predictor of prevalent coronary heart disease (OR = 2.07), and the prevalence of coronary heart disease markedly increases with MetS^27^. In addition, a recent meta-analysis by Mottillo and coworkers^28^ showed MetS associated with increased risk of cardiovascular disease (relative risk [RR] = 2.35), myocardial infarction (RR = 1.99), stroke (RR = 2.27), cardiovascular mortality (RR = 2.40), and all-cause mortality (RR = 1.58); another meta-analysis of longitudinal studies^10^ demonstrated a similar result with an overall pooled RR of 1.78 for incident cardiovascular events and death with MetS. The identification of people with MetS may provide opportunities to intervene at an earlier stage of cardiovascular disease and to extend life in the adult population^7^,^28^.

MetS is highly prevalent worldwide and affects approximately one-quarter of North Americans^29^,^30^. In Asia, the adjusted prevalence of MetS by the IDF criteria was 25.8% (men 23.1%, women 28.2%) among Asian Indians^31^, 12.8% (17.0%) among Mauritian Indian men (women)^21^, and 13.8% (2.5%) among Japanese men (women)^21^, respectively; and 24.2% (men 24.7%, women 23.8%) among Malays by the NCEP-ATPIII criteria^22^. In China, the prevalence of MetS was 9.8% to 18.1% for men and 11.4% to 28.0% for women by the IDF or NCEP-ATPIII criteria^18^,^20^,^32^,^33^,^34^. According to the MMRR criteria, the overall standardized prevalence of MetS among Shantou adults was 13.5% (men 14.2%, women 12.9%). Application of the prevalence rate to 2010 China census counts suggested that about 1.9 million Shantou natives have MetS. However, we should be cautious to directly compare our results with corresponding data from other research because the criteria used for determining MetS, the selection of the sample, and standardization and temporal differences affect prevalence estimation. In line with previous studies^29^,^31^,^32^, we found that the prevalence of MetS greatly increased with increasing age regardless of the definition used. The prevalence of MetS was higher for women than men among older adults but lower among younger adults, which is consistent with other surveys^21^,^35^.

Although the agreement of the MMRR with JCDCG and IDF criteria was “substantial”, the proportions of individual MetS components by the MMRR criteria are not exactly the same as those by the JCDCG or IDF criteria. From the Venn diagram, subjects with MetS defined by the 3 criteria have some overlap but not much. In the present study, 438 (9.6%) participants were identified as having MetS by all 3 criteria and 940 (20.5%) by any of the 3 criteria. The MMRR criteria could detect some subjects with MetS who could not be confirmed as having MetS by the JCDCG or IDF criteria.

As well, the cut-offs for MetS components differ slightly among the 3 criteria. For WC, the cut-off for central obesity was similar for men (89 or 90 cm) but slightly differed for women (80–85 cm). A 7.8-year follow-up study showed that the appropriate WC cut-offs for central obesity were 88 cm for men and 82 cm for women in the Chinese population^36^, and the cut-off points for WC (89 cm for men and 81 cm for women) by the MMRR criteria are closest to these results. Consistent evidence has demonstrated that Chinese men have a higher TG level than women^18^,^20^,^21^,^33^,^37^, so the cut-off for abnormal TG level should differ by gender, which was considered by the population-based MMRR criteria but not the IDF or JCDCG criteria. Notably, among the 3 criteria, reduced HDL-C level in women was detected with the highest frequency by the IDF criteria, because the highest cut-off point for HDL-C level (1.29 mmol/L) was used for identifying reduced HDL-C level, and such a cut-off may not be appropriate for Chinese people^20^,^37^. With the IDF criteria, the standardized prevalence of reduced HDL-C level among Shantou adults was surprisingly more than three-fold higher for women than me (40.0% vs 12.8%) n. Women with normal HDL-C levels will have a higher probability of having reduced HDL-C level by the IDF criteria, which increases false-positive observations. As compared with the JCDCG and IDF criteria, the MMRR criteria have higher cut-offs for BP and therefore produce a lower prevalence of elevated BP. The cut-off of FPG ≥ 6.1 mmol/L for hyperglycaemia by the JCDCG criteria is slightly high for Chinese people^1^,^17^, and we suggest decreasing the FPG cut-off to 5.6–5.8 mmol/L. Overall, the population-based MMRR criteria for diagnosing MetS are more appropriate for Shantou adults.

Several limitations in our study should be acknowledged. First, our study had sampling selection bias toward males. To avoid such bias, the MMRR criteria were established separately for men and women. Moreover, we standardized by sex and age for evaluating the prevalence of MetS with different weights. Nevertheless, a larger sample with a gender balance should be investigated in the future. Second, a measure of insulin resistance was not included as a component of MetS in the MMRR criteria because of its complicated test and costly price. It might be considered one of the components for the MMRR criteria in the future with regular testing.

MetS has become an important public health problem^20^. In the public health arena, the desirable way to reduce MetS is by lifestyle intervention, especially weight reduction, increased physical activity, and an anti-atherogenic diet^3^. Understanding the prevalence of MetS is critical for allocating health care resources and reducing the enormous economic burden of cardiovascular diseases to society^20^,^35^.

In conclusion, the newly established MMRR criteria of MetS agree substantially with the JCDCG and IDF criteria, but the recommended reference values and proportions for MetS components differ. The population-based MMRR criteria may be appropriate for diagnosing MetS in the Shantou population in China and the 95% MMRR model might be useful for generalization to other geographic regions. This study may help in better identifying people with MetS to reduce MetS-related disease.

## Additional Information

**How to cite this article**: Ding, Z. *et al.* Establishment and application of a new diagnostic definition of metabolic syndrome in the Shantou region of southern China. *Sci. Rep.* **6**, 22210; doi: 10.1038/srep22210 (2016).

## Figures and Tables

**Table 1:** Definitions of metabolic syndrome (MetS) by the MMRR, JCDCG, and IDF diagnostic criteria.

Components of MetS	MMRR		JCDCG		IDF	
	Men	Women	Men	Women	Men	Women
WC, cm	≥89	≥81	>90	>85	≥90	≥80
TG, mmol/L	≥1.73 or specific treatment of TG abnormality	≥1.64 or specific treatment of TG abnormality	≥1.70	≥1.70	≥1.7 or specific treatment of TG abnormality	≥1.7 or specific treatment of TG abnormality
HDL-C, mmol/L	≤1.07 or specific treatment of HDL-C abnormality	≤1.05 or specific treatment of HDL-C abnormality	<1.04	<1.04	<1.03 or specific treatment of HDL-C abnormality	<1.29 or specific treatment of HDL-C abnormality
BP, mmHg	SBP ≥ 138 or DBP ≥ 89 or treatment of previously diagnosed hypertension	SBP ≥ 136 or DBP ≥ 85 or treatment of previously diagnosed hypertension	SBP ≥ 130 or DBP ≥ 85	SBP ≥ 130 or DBP ≥ 85	SBP ≥ 130 or DBP ≥ 85 or treatment of previously diagnosed hypertension	SBP ≥ 130 or DBP ≥ 85 or treatment of previously diagnosed hypertension
FPG, mmol/L	≥ 5.8 or previously diagnosed type 2 diabetes	≥ 5.7 or previously diagnosed type 2 diabetes	≥ 6.1 or 2-h plasma glucose ≥ 7.8 or diagnosed diabetes	≥ 6.1 or 2-h plasma glucose ≥ 7.8 or diagnosed diabetes	≥ 5.6 or previously diagnosed type 2 diabetes	≥ 5.6 or previously diagnosed type 2 diabetes
Required	≥ 3 risk factors	≥ 3 risk factors	≥ 3 risk factors	≥ 3 risk factors	central obesity plus ≥ 2 other risk factors	central obesity plus ≥ 2 other risk factors

MMRR, multivariate medical reference range; JCDCG, Chinese Joint Committee for Developing Chinese Guidelines; IDF, International Diabetes Federation; WC, waist circumference; TG, triglycerides; HDL-C, high-density lipoprotein cholesterol; SBP/DBP, systolic/diastolic blood pressure; FPG, fasting plasma glucose.

**Table 2:** Characteristics of the study population in the Shantou region of southern China.

Variables	Total (n = 4,580)	Men (n = 3,062)	Women (n = 1,518)
Age, years	44.0 (36.0–57.0)	43.0 (35.0–54.0)	47.0 (37.0–60.0)^*^
WC, cm	81.0 (74.0–87.0)	82.8 (77.0–88.0)	76.0 (69.0–83.0)^*^
TG, mmol/L	1.27 (0.89–1.93)	1.35 (0.96–2.09)	1.08 (0.76–1.63)^*^
HDL-C, mmol/L	1.31 (1.13–1.50)	1.28 (1.11–1.46)	1.36 (1.16–1.58)^*^
SBP, mmHg	121.0 (110.0–134.4)	122.0 (112.0–134.0)	120.0 (110.0–135.0)^*^
DBP, mmHg	80.0 (70.0–85.0)	80.0 (72.0–86.0)	77.0 (70.0–82.0)^*^
FPG, mmol/L	5.07 (4.70–5.53)	5.07 (4.70–5.51)	5.08 (4.70–5.59)

Data are median (interquartile range [IQR]).

*P < 0.001 comparing women with men by the Mann–Whitney U test.

WC, waist circumference; TG, triglycerides; HDL-C, high-density lipoprotein cholesterol; SBP/DBP, systolic/diastolic blood pressure; FPG, fasting plasma glucose.

**Table 3:** Standardized prevalence of MetS and its individual components by the MMRR, JCDCG, and IDF criteria.

	MMRR (%)			JCDCG (%)			IDF (%)		
	Men^*^	Women^*^	Total^†^	Men^*^	Women^*^	Total^†^	Men^*^	Women^*^	Total^†^
Individual components of MetS									
Elevated WC	21.5^¶^	28.7^‡¶^	25.0^¶^	15.9^§^	14.3^§^	15.1^§^	18.7	32.5^‡^	25.5
Elevated TG	30.4^‡^	19.8	25.2	31.1^‡^	18.5	24.9	31.1^‡^	18.5	24.9
Reduced HDL-C	19.2^‡¶^	13.9^¶^	16.6^¶^	13.9^‡§^	11.2^§^	12.6^§^	12.8	40.0^‡^	26.3
Elevated SBP and/or DBP	25.3^¶^	24.5^¶^	24.9^¶^	42.5^‡§^	29.6^§^	36.1^§^	42.5^‡^	29.6	36.1
Elevated FPG	14.4^¶^	16.1^¶^	15.2^¶^	10.3^§^	9.7^§^	10.0^§^	19.6	19.7	19.7
MetS	14.2^¶^	12.9^¶^	13.5^¶^	13.8^‡^	9.4^§^	11.6^§^	11.2	15.7^‡^	13.4
Number of metabolic abnormalities									
0	40.1	43.2	41.6	36.5	51.2	43.8	36.7	32.7	34.7
1	28.7	28.4	28.5	31.5	26.8	29.2	32.5	40.1	36.2
2	17.1	15.6	16.3	18.2	12.6	15.4	20.6	16.4	18.6
3	9.6	8.3	8.9	9.7	6.6	8.2	8.6	8.3	8.5
4	3.8	4.2	4.0	3.6	2.5	3.1	1.6	2.5	2.0
5	0.8	0.4	0.6	0.5	0.3	0.4	0	0	0

*Age-standardized prevalence rates.

†Age-standardized and sex-standardized prevalence rates. Standardization was conducted with the 2010 Chinese National Census Population by the direct method.

^‡^*P* < 0.001, significantly higher than for the other sex.

^¶^*P* < 0.001, the standardized proportions were significantly different among the MMRR, JCDCG, and IDF criteria.

^§^*P* < 0.001 comparing the JCDCG with the MMRR criteria. ^ÿ^P < 0.001 comparing the IDF with the MMRR criteria.

MetS, metabolic syndrome; MMRR, multivariate medical reference range; JCDCG, Chinese Joint Committee for Developing Chinese Guidelines; IDF, International Diabetes Federation; WC, waist circumference; TG, triglycerides; HDL-C, high-density lipoprotein cholesterol; SBP/DBP, systolic/diastolic blood pressure; FPG, fasting plasma glucose.

**Table 4:** Agreement between the MMRR and JCDCG, and MMRR and IDF criteria.

Criteria	MMRR						
	Observed agreement (%)	Sensitivity (%)	Specificity (%)	PPV (%)	NPV (%)	Kappa index (95% CI)	Judgment for agreement
JCDCG							
Total	94.48	87.60	95.62	76.88	97.89	0.79 (0.76, 0.81)^*^	substantial
Men	94.94	85.31	96.65	81.95	97.36	0.81 (0.78, 0.84)^*^	almost perfect
Women	93.54	93.16	93.60	67.56	98.96	0.75 (0.70, 0.80)^*^	substantial
IDF							
Total	91.88	76.80	94.57	71.64	95.80	0.69 (0.66, 0.72)^*^	substantial
Men	92.33	82.41	93.73	65.15	97.40	0.68 (0.65, 0.72)^*^	substantial
Women	90.97	69.97	96.43	83.59	92.52	0.71 (0.66, 0.75)^*^	substantial

*P < 0.001 testing the null hypothesis that the extent of agreement is same as random (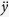 = 0).

MMRR, multivariate medical reference range; JCDCG, Chinese Joint Committee for Developing Chinese Guidelines; IDF, International Diabetes Federation; PPV, positive predictive value; NPV, negative predictive value; 95% CI, 95% confidence interval.

## References

[b1] Grundy S. M. *et al.* Diagnosis and management of the metabolic syndrome: an American Heart Association/National Heart, Lung, and Blood Institute Scientific Statement. Circulation 112, 2735–2752 (2005). 16157765 10.1161/CIRCULATIONAHA.105.169404

[b2] Alberti K. G., Zimmet P. & Shaw J. Metabolic syndrome—a new world-wide definition. A Consensus Statement from the International Diabetes Federation. Diabet Med 23, 469–480 (2006). 16681555 10.1111/j.1464-5491.2006.01858.x

[b3] Grundy S. M. Metabolic syndrome: connecting and reconciling cardiovascular and diabetes worlds. J Am Coll Cardiol 47, 1093–1100 (2006). 16545636 10.1016/j.jacc.2005.11.046

[b4] Stern M. P., Williams K., Gonzalez-Villalpando C., Hunt K. J. & Haffner S. M. Does the metabolic syndrome improve identification of individuals at risk of type 2 diabetes and/or cardiovascular disease? Diabetes care 27, 2676–2681 (2004). 15505004 10.2337/diacare.27.11.2676

[b5] Sattar N. *et al.* Can metabolic syndrome usefully predict cardiovascular disease and diabetes? Outcome data from two prospective studies. Lancet 371, 1927–1935 (2008). 18501419 10.1016/S0140-6736(08)60602-9

[b6] Iribarren C. *et al.* Metabolic syndrome and early-onset coronary artery disease: is the whole greater than its parts? J Am Coll Cardiol 48, 1800–1807 (2006). 17084253 10.1016/j.jacc.2006.03.070

[b7] McNeill A. M. *et al.* The metabolic syndrome and 11-year risk of incident cardiovascular disease in the atherosclerosis risk in communities study. Diabetes care 28, 385–390 (2005). 15677797 10.2337/diacare.28.2.385

[b8] Chang J. J. *et al.* Differences in prevalence and severity of coronary artery disease by three metabolic syndrome definitions. Can J Cardiol 28, 208–214 (2012). 22244771 10.1016/j.cjca.2011.10.016

[b9] Vinluan C. M., Zreikat H. H., Levy J. R. & Cheang K. I. Comparison of different metabolic syndrome definitions and risks of incident cardiovascular events in the elderly. Metabolism 61, 302–309 (2012). 21840552 10.1016/j.metabol.2011.07.002 PMC3218249

[b10] Gami A. S. *et al.* Metabolic syndrome and risk of incident cardiovascular events and death: a systematic review and meta-analysis of longitudinal studies. J Am Coll Cardiol 49, 403–414 (2007). 17258085 10.1016/j.jacc.2006.09.032

[b11] Lakka H. M. *et al.* The metabolic syndrome and total and cardiovascular disease mortality in middle-aged men. JAMA 288, 2709–2716 (2002). 12460094 10.1001/jama.288.21.2709

[b12] Alberti K. G. & Zimmet P. Z. Definition, diagnosis and classification of diabetes mellitus and its complications. Part 1: diagnosis and classification of diabetes mellitus provisional report of a WHO consultation. Diabet Med 15, 539–553 (1998). 9686693 10.1002/(SICI)1096-9136(199807)15:7<539::AID-DIA668>3.0.CO;2-S

[b13] Executive Summary of The Third Report of The National Cholesterol Education Program (NCEP) Expert Panel on Detection, Evaluation, And Treatment of High Blood Cholesterol In Adults (Adult Treatment Panel III). *JAMA* **285**, 2486-2497 (2001). 10.1001/jama.285.19.2486 11368702

[b14] Third Report of the National Cholesterol Education Program (NCEP) Expert Panel on Detection, Evaluation, and Treatment of High Blood Cholesterol in Adults (Adult Treatment Panel III) final report. *Circulation* **106**, 3143–3421 (2002). 12485966

[b15] Alberti K. G., Zimmet P. & Shaw J. The metabolic syndrome—a new worldwide definition. Lancet 366, 1059–1062 (2005). 16182882 10.1016/S0140-6736(05)67402-8

[b16] International Diabetes Federation. The IDF consensus worldwide definition of the metabolic syndrome. Brussels, Belgium: International Diabetes Federation (2006).

[b17] Joint Committee for Developing Chinese guidelines on Prevention and Treatment of Dyslipidemia in Adults. [Chinese guidelines on prevention and treatment of dyslipidemia in adults]. Chin J Cardiol 35, 390–419 (2007). 17711682

[b18] Wang C. *et al.* The metabolic syndrome increased risk of cardiovascular events in Chinese—a community based study. Int J Cardiol 139, 159–165 (2010). 19046783 10.1016/j.ijcard.2008.10.012

[b19] Yi D. *et al.* Reduced risk of dyslipidaemia with oolong tea consumption: a population-based study in southern China. Br J Nutr 111, 1421–1429 (2014). 24229494 10.1017/S0007114513003644

[b20] Gu D. *et al.* Prevalence of the metabolic syndrome and overweight among adults in China. Lancet 365, 1398–1405 (2005). 15836888 10.1016/S0140-6736(05)66375-1

[b21] Prevalence of the metabolic syndrome in populations of Asian origin. Comparison of the IDF definition with the NCEP definition. Diabetes Res Clin Pract 76, 57–67 (2007). 17010470 10.1016/j.diabres.2006.07.020

[b22] Tan C. E., Ma S., Wai D., Chew S. K. & Tai E. S. Can we apply the National Cholesterol Education Program Adult Treatment Panel definition of the metabolic syndrome to Asians? Diabetes care 27, 1182–1186 (2004). 15111542 10.2337/diacare.27.5.1182

[b23] Hekking M., Lindemans J. & Gelsema E. S. A computer program for constructing multivariate reference models. Comput Methods Programs Biomed 53, 191–200 (1997). 9230454 10.1016/s0169-2607(97)00018-7

[b24] Koduah M., Iles T. C. & Nix B. J. Centile charts I: new method of assessment for univariate reference intervals. Clin Chem 50, 901–906 (2004). 15016727 10.1373/clinchem.2003.023762

[b25] Boyd J. C. & Lacher D. A. The multivariate reference range: an alternative interpretation of multi-test profiles. Clin Chem 28, 259–265 (1982). 7055945

[b26] Landis J. R. & Koch G. G. The measurement of observer agreement for categorical data. Biometrics 33, 159–174 (1977). 843571

[b27] Alexander C. M., Landsman P. B., Teutsch S. M. & Haffner S. M. NCEP-defined metabolic syndrome, diabetes, and prevalence of coronary heart disease among NHANES III participants age 50 years and older. Diabetes 52, 1210–1214 (2003). 12716754 10.2337/diabetes.52.5.1210

[b28] Mottillo S. *et al.* The metabolic syndrome and cardiovascular risk a systematic review and meta-analysis. J Am Coll Cardiol 56, 1113–1132 (2010). 20863953 10.1016/j.jacc.2010.05.034

[b29] Ford E. S., Giles W. H. & Dietz W. H. Prevalence of the metabolic syndrome among US adults: findings from the third National Health and Nutrition Examination Survey. JAMA 287, 356–359 (2002). 11790215 10.1001/jama.287.3.356

[b30] Beltran-Sanchez H., Harhay M. O., Harhay M. M. & McElligott S. Prevalence and trends of metabolic syndrome in the adult U.S. population, 1999-2010. J Am Coll Cardiol 62, 697–703 (2013). 23810877 10.1016/j.jacc.2013.05.064 PMC3756561

[b31] Deepa M., Farooq S., Datta M., Deepa R. & Mohan V. Prevalence of metabolic syndrome using WHO, ATPIII and IDF definitions in Asian Indians: the Chennai Urban Rural Epidemiology Study (CURES-34). Diabetes Metab Res Rev 23, 127–134 (2007). 16752431 10.1002/dmrr.658

[b32] Hwang L. C., Bai C. H. & Chen C. J. Prevalence of obesity and metabolic syndrome in Taiwan. J Formos Med Assoc 105, 626–635 (2006). 16935763 10.1016/S0929-6646(09)60161-3

[b33] Liu J. *et al.* Ethnic-specific criteria for the metabolic syndrome: evidence from China. Diabetes care 29, 1414–1416 (2006). 16732037 10.2337/dc06-0481

[b34] Cheung B. M. *et al.* Development of diabetes in Chinese with the metabolic syndrome: a 6-year prospective study. Diabetes care 30, 1430–1436 (2007). 17337491 10.2337/dc06-1820

[b35] Zhao Y. *et al.* Prevalence and determinants of metabolic syndrome among adults in a rural area of Northwest China. PloS one 9, e91578 (2014). 24614618 10.1371/journal.pone.0091578 PMC3948893

[b36] Ye Y. *et al.* Identification of waist circumference cutoffs for abdominal obesity in the Chinese population: a 7.8-year follow-up study in the Shanghai urban area. Int J Obes (Lond) 33, 1058–1062 (2009). 19581913 10.1038/ijo.2009.134

[b37] Yang W. *et al.* Serum lipids and lipoproteins in Chinese men and women. Circulation 125, 2212–2221 (2012). 22492668 10.1161/CIRCULATIONAHA.111.065904

